# Patient Satisfaction and Quality of Life in DIEAP Flap versus Implant Breast Reconstruction

**DOI:** 10.1155/2015/405163

**Published:** 2015-11-16

**Authors:** Rossella Sgarzani, Luca Negosanti, Paolo Giovanni Morselli, Veronica Vietti Michelina, Luigi Maria Lapalorcia, Riccardo Cipriani

**Affiliations:** ^1^Plastic Surgery Department, Sant'Orsola-Malpighi Hospital, University of Bologna, Via Massarenti 9, 40138 Bologna, Italy; ^2^Plastic Surgery Department, Asl 1 of Umbria, Citta di Castello, Località Chioccolo, 06012 Perugia, Italy

## Abstract

The psychological impact of breast reconstruction has widely been described, and multiple studies show that reconstruction improves the well-being and quality of life of patients. In breast reconstruction, the goal is not only the morphological result, but mainly the patient's perception of it. The objective of our study is to compare the physical and psychosocial well-being and satisfaction concerning the body image of patients who had reconstruction with breast implants to those of patients who had reconstruction with deep inferior epigastric artery perforator flaps. Our results demonstrated a similar quality of life between the two groups, but the satisfaction level was significantly higher in patients who had reconstruction with autologous tissue. Feedback from patients who have already received breast reconstruction may be useful in the decision-making process for future patients and plastic surgeons, enabling both to choose the reconstructive technique with the best long-term satisfaction.

## 1. Introduction

Many studies evaluate the outcomes of breast reconstruction, but only a few examine the satisfaction of patients who received breast reconstruction with autologous tissues [1].

Despite the continuous increase of early diagnosis and conservative treatments for breast cancer, in 25% of patients, a mastectomy remains the gold standard [2, 3]. This mutilating procedure is a traumatizing event, and many psychological disorders have been linked to this surgery in the literature [4–9].

The role of breast reconstruction after a mastectomy has been widely demonstrated [10], and multiple studies have shown that breast reconstruction improves patients' well-being and quality of life [11, 12].

Several reports show that women who undergo breast reconstruction after mastectomy have less psychological distress and have an improved quality of life compared to women who refuse any reconstructive option [13, 14].

The aim of the present study is to evaluate the physical and psychosocial well-being of patients who underwent breast reconstruction as well as compare the long-term satisfaction of patients who underwent reconstruction with implants with patients who underwent reconstruction with a Deep Inferior Epigastric Artery Perforator (DIEAP) flap.

These two techniques represent the gold standards in breast reconstruction.

The present study is based on a self-evaluating questionnaire to acquire new data concerning the personal satisfaction of patients who have already undergone breast reconstruction and analyze the feelings of patients concerning different reconstruction phases.

## 2. Materials and Methods

Retrospective observational single center study (S. Orsola-Malpighi Hospital, Bologna, Italy) was performed.

The inclusion criteria of the study were as follows:

- adult patients;
- unilateral mastectomy for breast cancer or prophylaxis;
- immediate or delayed breast reconstruction with expander/implant or DIEAP flap;
- reconstruction performed between 2007 and 2011 (in order to have a minimum follow-up time of 36 months).

Patients who met these criteria were identified through our hospital database and were contacted by telephone. They were presented with the opportunity to take part in the study and were offered an appointment at the Plastic Surgery Outpatient Clinic to independently complete the questionnaire; the aim of the study and the average time to complete the questionnaire were explained.

We followed the Dillman method to maximize the percentage of responders including subsequent calls to nonresponders [15].

We contacted 129 patients by telephone, and 87 of them answered.

Sixty-three patients agreed to participate in the study; 4 patients deceased and 20 refused.

Each of the 63 patients participating in the study was welcomed in the Plastic Surgery Outpatient Clinic by a staff member. The purpose of the study was reemphasized, they were informed that all data would remain anonymous, and they signed an informed consent and a sensitive data consent. The questionnaire was delivered to the patient alone, so it could be completed independently.

### 2.1. Self-Evaluation Questionnaire

The Breast-Q questionnaire (Memorial Sloan-Kettering Cancer Center and The University of British Columbia, 2006, all rights reserved), designed for patients undergoing breast surgery and specifically for patients undergoing breast reconstruction, was used [16, 17].

The conceptual framework of the questionnaire is formed by two main domains: one related to the quality of life (investigating physical, psychosocial, and sexual well-being) and the other regarding satisfaction (satisfaction with the breast, overall outcome, and the care process).

The average time to administer the questionnaire was 15–20 minutes.

### 2.2. Data Analysis

The population was divided into two groups. Group A included 33 patients (52.4%) who had reconstruction with autologous tissue (all of these procedures were DIEAP flaps performed by the same senior surgeon). Group B included 30 patients (47.6%) who had reconstruction using expanders and implants (these surgeries were performed by four senior consultants).

The obtained data were reported in Excel (Microsoft Corp., Redmond, WA, USA) and were analyzed using SPSS statistical software package version 17.0 (SPSS Inc., Chicago, IL, USA).

The average, median, and mode were assessed as position indexes considering the inherent characteristics of our group of responders that did not show a Gaussian distribution for most of the parameters. The evaluation of the median and quartiles was considered more appropriate as the values of skewness and kurtosis were far from 0.

Mann-Whitney and Student's *t*-tests for parametric variables were used to compare the two groups, that is, DIEAP flap and expander/implant; the Wilcoxon test was also used for the same assessments.

Correlation studies were performed using nonparametric Spearman's rho.

Pearson Chi-Square and Fisher's exact tests were used to determine the association between a dependent variable and an independent one (a *P* value < 0.05 was considered to be statistically significant).

## 3. Results

A total of 129 patients were contacted by telephone, and 87 of them answered (67%).

Of these, 63 patients agreed to take part in the study. The percentage of responders was 72.4%; this is comparable to other studies [18].

The patients were divided into two groups. Group A included 33 patients (52.4%) who underwent reconstruction with DIEAP flaps, and group B included 30 patients (47.6%) who underwent reconstruction with expanders/implants.

The mean age was 53.03 years (ranging between 31 and 74 years); 17.5% were unmarried, 66.7% were married, 11.1% were divorced, and 4.8% were widowed.

We evaluated the differences between the two groups; no statistically significant differences in age (*P* < 0.432), marital status (*P* < 0.087), or follow-up time (*P* < 0.922) were found (Table 1).

The evaluation of the collected data demonstrated a good level of satisfaction with the reconstructed breast (3.1038 out of 4) and a high satisfaction with the overall result (2.714 out of 3). These results emphasize the positive value of breast reconstruction after mastectomy.

**Figure 1:**
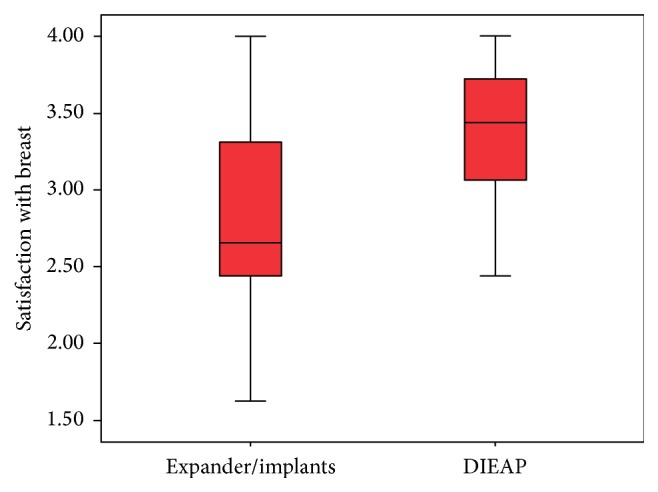
Box plot showing satisfaction with the reconstructed breast.

In all subscales, patients undergoing breast reconstruction with a DIEAP flap reported higher scores, but the score reached statistical significance only in satisfaction with the reconstructed breast (Figure 1) (implants 2.8393 out of 4; DIEAP 3.3427 out of 4) (*P* < 0.002), overall result (implants 2.6667 out of 3; DIEAP 2.7576 out of 3) (*P* < 0.041), and nipple areola complex (NAC) reconstruction (implants 2.6471 out of 4; DIEAP 3.2208 out of 4) (*P* < 0.007) (Table 2).

Concerning the sexual well-being scale, DIEAP flap patients were more satisfied than the expander/implant patients, 3.2644 versus 3.1358, respectively, but this difference did not reach statistical significance (*P* < 0.699). Patients undergoing DIEAP flap reconstruction reported higher scores in psychosocial and physical well-being, which did not reach statistical significance (*P* < 0.121 and *P* < 0.214, resp.).

Patients undergoing DIEAP flap breast reconstruction reported greater satisfaction with the medical team (*P* < 0.027), which was statistically significant, in addition to greater satisfaction with the surgeon (*P* < 0.253) and the administrative team (*P* < 0.232).

**Figure 2:**
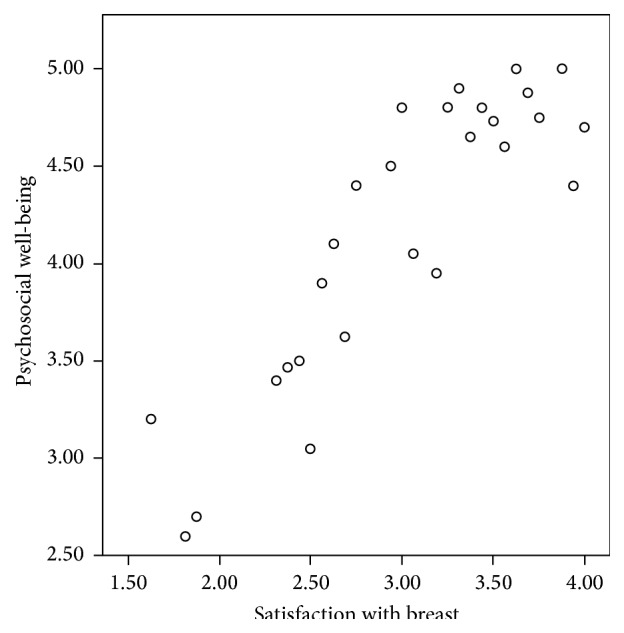
Correlation between reconstructed breast satisfaction and psychosocial well-being.

Satisfaction with the reconstructed breast correlates with overall satisfaction and with psychosocial and sexual well-being, reaching statistical significance in both groups (all *P* < 0.000) (Figure 2).

Another finding was that satisfaction about the information given preoperatively was linked to satisfaction with the surgeon (*P* < 0.002 in both groups) and the medical team (*P* < 0.002 for group B; *P* < 0.035 for group A).

We assessed how the follow-up time affected patient satisfaction, but no statistically significant differences were found. The average follow-up time was 3.1587 years (ranging from 3 to 6 years). We compared the two groups using the Mann-Whitney test, which did not show a statistically significant difference (*P* < 0.922).

Regarding NAC reconstruction among the 63 patients who participated in the study, only 47 had undergone NAC reconstruction (74.6%). Three of the patients had not yet received the tattoo to match the color of the contralateral areola at the time of this study.

All patients received a nipple reconstruction using the same technique (star-flap), avoiding bias resulting from different techniques.

**Figure 3:**
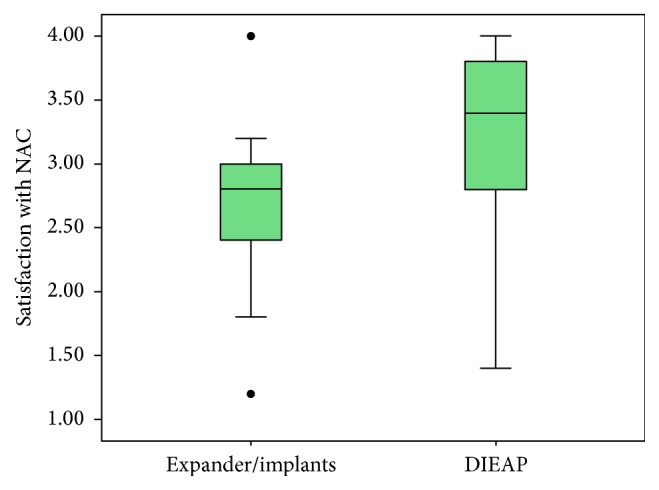
Box plot showing satisfaction with the reconstructed NAC.

With NAC, we found greater satisfaction in patients undergoing autologous tissue reconstruction (*P* < 0.007) (Figure 3). We assessed the satisfaction related to shape, general appearance, naturalness, color, and NAC projection in the two groups. We dichotomized responses into “satisfied” for patients who provided values of 3 (somewhat satisfied) or 4 (very satisfied) and “dissatisfied” for values of 1 (somewhat dissatisfied) or 2 (very dissatisfied).

In the expander/implant group, 68.4% were satisfied with the shape and appearance; in the DIEAP flap group, 85.7% were satisfied with those metrics. This difference did not reach statistical significance (*P* < 0.155).

A significant difference between the two groups was found regarding the NAC naturalness. Of the patients reconstructed with a DIEAP flap, 78.7% declared themselves satisfied, compared with 31.6% in the expander/implant group. The significance was assessed using Pearson's Chi-Square (*P* < 0.001) and Fisher's exact tests (*P* < 0.002). Patients whose breasts were reconstructed using a DIEAP flap were also more satisfied with the NAC projection (85.7% versus 78.9% in second group).

We also found a statistically significant correlation between satisfaction with the nipple naturalness and the marital status of the patient; single women showed a greater significance than the married patients (*P* < 0.005 for single and *P* < 0.033 for married women).

Correcting the natural satisfaction of the nipple in relation to marital status, women reconstructed using a DIEAP flap were 2 to 33 times more satisfied than the others; the odds ratio was corrected according to a Mantel-Haenszel value of 8.438 (confidence limits 95%) (2.138–33.303) (*P* < 0.002).

## 4. Discussion

The decision-making process of a patient undergoing breast cancer surgery is very complex. Many initiatives have been developed recently to provide patients with correct and complete information on reconstructive and nonreconstructive options, such as Breast Reconstruction Awareness Day [19].

Identifying the best source of information for the patient is one question that remains. Medical and paramedical staff in hospitals can provide objective details on surgical options, surgical time needed for each procedure, risks, complication rates, postoperatory recovery times, and hospitalizations [20].

The Internet is a very useful tool for patients to seek information, but not all the information on the Internet is reliable and not all of it meets the expectations of the patients [21]. Many surgical centers currently provide their patients with booklets [22] or website references where they can find correct information [23, 24].

Other patient experiences can be another important source of information. The possibility to provide objective information about other patients' satisfaction levels following different procedures is very useful [25].

Perceived outcome information can be collected through self-evaluating questionnaires assessing the quality of life and patient satisfaction regarding several aspects, including appearance, psychological well-being, and sexual well-being.

Breast-Q, the questionnaire administered in the present study, is designed for patients undergoing breast surgery and reconstruction. It was developed following qualitative and quantitative psychometric methods and meets the international criteria for the outcome assessment [26, 27].

In our study, we decided to include only unilateral breast reconstruction because we believe that the most important aspect of a reconstruction is symmetry. Symmetry influences the posture of the patient and her confidence in her appearance. A bilateral reconstruction with an implant or autologous tissue is more often symmetrical both in the immediate postoperation period and in the long term. The real challenge is reaching and maintaining symmetry in unilateral reconstructions, and our study aims to investigate this aspect.

Through statistical analysis, our results showed that patients who underwent autologous tissue reconstruction were more satisfied than those who received an expander/implant reconstruction, reaching statistical significance in satisfaction with the breast, overall outcome, and NAC reconstruction.

These data confirm previous reports in the literature, with a general consensus suggesting that patients whose breasts are reconstructed using autologous tissue are more satisfied [18, 28, 29].

In this study, only DIEAP flap breast reconstruction was considered in the autologous tissue reconstructive method because the Transverse Rectus Abdominis Muscle flap or the Latissimus Dorsi flap are not performed in our Plastic Surgery Division because of donor site morbidity.

Yueh et al. [18] showed that patients who underwent reconstruction using perforator flaps are more likely to have a higher overall satisfaction compared to those who underwent reconstruction using nonperforator flaps (82.7% versus 65.8%; *P* < 0.002).

The same authors showed that patients undergoing autologous tissue reconstruction were more satisfied with the reconstructed breast than patients receiving implants. Among women who had reconstruction with autologous tissue, those who received a flap taken from the abdominal region were more satisfied than patients who underwent reconstruction with a Latissimus Dorsi flap. When comparing patients who received either TRAM or DIEAP flaps, the difference was no longer statistically significant, although the difference between those two methods in terms of donor site morbidity is well described [30].

Other authors reported satisfaction with breast reconstruction using an abdominal flap but also dissatisfaction with the donor site [31].

We did not include any Superficial Inferior Epigastric Artery (SIEA) flap reconstruction in the study in order to have a uniform group of autologous reconstructed patients and avoid bias linked to the “easier” postoperative period and donor site recovery of the SIEA flap compared to DIEAP flap reconstruction.

In our study, 91% of the DIEAP patients perceived their reconstructed breast as a natural part of their own body, while only 40% of patients who underwent prosthetic reconstruction stated the same.

In our study, we did not demonstrate a significant difference in satisfaction with increasing follow-up time. This evaluation had been made in relation to a previous work published by Hu et al. [32] in which the authors stressed that both breast implants and autologous tissue reconstruction would experience an “aging” process, resulting in different long-term complications that can variably influence the aesthetic result.

The authors noted that patients who underwent TRAM, compared to patients who underwent expander/implant reconstruction, showed greater long-term aesthetic satisfaction. The satisfaction reduction in patients who underwent breast reconstruction using an expander/implant could be related to the high incidence of complications and reoperations that this technique requires [33–35]. Women who undergo reconstruction using silicone gel implants have a 20% risk of developing grade III or IV Baker capsular contracture [36] and a 30% risk of having to remove or replace the prosthesis, resulting in an overall reoperation rate of 45–50% [37].

Interestingly, our result showed no significant reduction in satisfaction over time in both groups, and this allows us to comment that patient perception of the reconstruction is a very complex process in the elaboration of a new body image that is not only correlated to actual symmetry.

It is important to note that our minimal follow-up time of 36 months might be considered insufficient to show the long-term differences between implants and autologous tissues. However, with this timing, we were able to demonstrate that, already at 3 years, autologous breast reconstruction is more satisfactory than implant reconstruction in most of the parameters.

In the quality of life evaluation, we found that DIEAP flap patients reported higher scores in all aspects, but these scores did not reach statistical significance for psychosocial well-being (*P* < 0.121), sexual well-being (*P* < 0.699), or physical well-being (*P* < 0.214).

Patients undergoing a DIEAP flap for breast reconstruction reported statistically significantly greater satisfaction with the medical team (*P* < 0.027) and greater satisfaction with the surgeon (*P* < 0.253) and the administrative team (*P* < 0.232).

Another aspect specifically investigated in our study is the NAC reconstruction.

Although not all patients decide to proceed with NAC reconstruction, several psychological benefits for this reconstruction have been demonstrated in the literature [32]. In our study, 47 of the 63 women (74.6%) underwent nipple reconstruction; three had not yet completed the process of reconstruction or had avoided the intradermal tattoo of the areola.

We showed a statistically significant difference regarding satisfaction with the NAC between the two groups that were studied. Women who underwent breast reconstruction using autologous tissue are, according to our data, more satisfied with their nipple areola complex than those in the implant group.

Our study showed that satisfaction with the NAC projection depended on marital status. Single women were significantly (*P* < 0.005 versus *P* < 0.033) more satisfied with the NAC projection as opposed to women who did not undergo NAC reconstruction, a figure which may correlate with the need for these patients to relate to new partners.

In general, by correcting the satisfaction as a function of marital status, women whose breasts were reconstructed using DIEAP flaps were 2 to 33 times more satisfied with the NAC projection, compared with patients receiving implants, with an odds ratio of 8.438, corrected using Mantel-Haenszel (95%) (2.138–33.303) (*P* < 0.002).

As evidenced by Handel [33], a predictable outcome in the long term is the loss of projection, although this phenomenon is difficult to quantify. A reduction of the average nipple projection of at least 50% must be considered in the reconstruction; this flattening occurs especially in the first months after surgery.

The total and partial loss of nipple sensitivity, investigated in previous research [38], was mentioned by many women as a limiting factor that superseded the result of the reconstruction itself.

Satisfaction concerning the information given preoperatively was linked to satisfaction with the surgeon (*P* < 0.002 in both groups) and the medical team (*P* < 0.002 for the group expander prostheses; *P* < 0.035 for the DIEAP group). This result emphasizes the central role of the information process, which makes a more accurate and aware choice possible and allows for greater satisfaction in the postoperative phases.

Limitations are present in our study. A selection bias is unavoidable because patients cannot be randomized to the types of surgery or reconstruction they received. It appears impossible to control different personal characteristics of patients, such as adversity to risks or personality traits. Patients choosing reconstruction with implants may be systematically different from patients choosing reconstruction using autologous tissue (DIEAP flap), which could affect the results.

Another bias can be linked to responders. It is more likely that only patients who were very satisfied or very dissatisfied with the result decided to participate in the study.

This is a retrospective study, and it has a recall bias; patients were asked to remember different details of their reconstruction process, which may have taken place up to 6 years earlier.

All surgeries in group A were performed by the same surgeon, while patients in group B underwent surgery performed by 4 different consultants in our department who used the same technique. The use of different surgeons could have also affected the results concerning satisfaction related to the surgeon and the information process.

To improve the reliability of the study, a prospective study in which patients are subjected to self-evaluation both preoperatively and postoperatively may be undertaken.

We believe that the high response rate made our data reliable.

Further studies must be developed to understand the different components that work together and affect the overall satisfaction of patients undergoing reconstructive breast surgery.

## 5. Conclusion

Our results demonstrated an overall higher satisfaction in patients who underwent unilateral breast reconstruction using autologous tissue even 3 years after reconstruction, with a comparable quality of life between the autologous tissue and expander/implant groups.

Feedback from patients who have already gone through the difficult choices related to mastectomy and breast reconstruction may be useful in guiding future patients.

## Figures and Tables

**Table 1:** Demographical data of the two groups, showing no statistically significant differences between them.

	DIEAP	Expander/implant	*P*
Number of patients	33 (52.4%)	30 (47.6%)	
Age	52.45	53.7	0.432
(Range)	(from 32 to 74)	(from 31 to 71)	
Marital status			
Married	22	20	0.087
Unmarried	7	4	
Separated	0	4	
Divorced	1	2	
Widowed	3	0	
Follow-up time	3.39	3.17	0.922
(Range)	(from 1 to 6 years)	(from 1 to 5 years)	

**Table 2:** The statistical significance of differences between the two groups. The Mann-Whitney and Wilcoxon nonparametric tests allow comparing samples without a normal distribution.

	Reconstructed breast	Overall outcome	Psychosocial well-being	Sexual well-being	Physical well-being	NAC	Information	Surgeon	Medical team	Administrative team
*U* Mann-Whitney	225,500	350,000	383,000	368,000	390,500	112,000	284,000	419,500	358,000	412,000
*W* Wilcoxon	631,500	815,000	848,000	746,000	951,500	265,000	635,000	884,500	823,000	847,000
*Z*	−3,169	−2,004	−1,549	−0,386	−1,244	−2,721	−1,744	−1,143	−2,219	−1,194
*P*	0,002	0,041	0,121	0,699	0,214	0,007	0,081	0,253	0,027	0,232
